# Site-Specific Regulation of Sulfatase and Aromatase Pathways for Estrogen Production in Endometriosis

**DOI:** 10.3389/fmolb.2022.854991

**Published:** 2022-05-03

**Authors:** Katiane de Almeida Da Costa, Helena Malvezzi, Cristine Dobo, Rosa Maria Neme, Renée Zon Filippi, Thiago Pinheiro Arrais Aloia, Elisa Rampazo Prado, Juliana Meola, Carla de Azevedo Piccinato

**Affiliations:** ^1^ Hospital Israelita Albert Einstein, São Paulo, Brazil; ^2^ Department of Clinical Pathology, Hospital Israelita Albert Einstein, São Paulo, Brazil; ^3^ Centro de Endometriose São Paulo, Av. República Do Líbano, São Paulo, Brazil; ^4^ Department of Gynaecology & Obstetrics, School of Medicine of Ribeirão Preto, University of São Paulo, Ribeirão Preto, Brazil

**Keywords:** aromatase, *CYP19A1*, STS, HSD17B1, estradiol, stromal cells, glandular cells, endometrium

## Abstract

Endometriosis is a highly prevalent gynecological disease characterized by lesions in different sites. Regulation of specific estrogen pathways may favor the formation of distinct microenvironments and the progression of endometriosis. However, no study has simultaneously evaluated the gene and protein regulation of the main estrogen-synthesizing enzymes in endometriosis. Thus, our goals were to study the relationship between gene and protein expression of aromatase (*CYP19A1* or ARO), steroid sulfatase (STS), and hydroxysteroid 17-beta dehydrogenase (HSD17B1) in superficial (SUP), ovarian (OMA), and deep infiltrating (DIE) endometriotic lesion sites as well as in the eutopic endometrium of patients with (EE) and without (control) endometriosis in the same and large cohort of patients. The site-specific expression of these enzymes within different cells (glandular and stromal components) was also explored. The study included 108 patients surgically diagnosed with endometriosis who provided biopsies of EE and endometriotic lesions and 16 disease-free patients who collected normal endometrium tissue. Our results showed that *CYP19A1* was detected in all endometriosis tissues and was in higher levels than in control. Unique patterns of the *STS* and *HSD17B1* levels showed that they were most closely regulated in all tissues, with manifestation at greater levels in DIE compared to the other endometriotic lesion sites, OMA and SUP. Gene and protein expression of ARO, STS, and HSD17B1 occurred at different rates in endometriotic sites or EE. The distinctive levels of these estrogen-synthesizing enzymes in each endometriotic site support the hypothesis of a tissue microenvironment that can both influence and be influenced by the expression of different estrogenic pathways, locally affecting the availability of estrogen needed for maintenance and progression of endometriotic lesions.

## Introduction

Endometriosis is the most prevalent gynecological disease in women of reproductive age (Taylor et al., 2021), and it is an important worldwide public health problem (Olive and Schwartz, 1993; Signorile and Baldi, 2010). Endometriosis is characterized by endometrial tissue localized outside the uterus (ectopic lesions), usually at several sites, that causes a chronic inflammatory process predisposing to adhesion formation (Jackson and Telner, 2006; Vermeulen et al., 2021). The current estimation of endometriosis prevalence is around 10–15% of women of childbearing age (Eskenazi and Warner, 1997; Giudice and Kao, 2004) of whom 20–50% present infertility-related symptoms (Smolarz et al., 2021).

Intracrinology of endometriosis debates on the balance between estrogen biosynthesis and metabolism that determines local estrogen availability in the tissues (Piccinato et al., 2018a). Evidence of this mechanism was revealed by data showing that the levels of estradiol and estrone remained constant in the lesions, despite the menstrual cyclical changes in circulating levels and the eutopic endometrium (Huhtinen et al., 2012). Such features indicate that, within the lesions, there are intrinsic regulatory pathways, which maintain high tissue availability of estrogen necessary for disease progression. As reported in previous studies, there are controversial data on gene regulation and protein levels of estradiol-synthesizing enzymes. Some studies have shown that, in endometriotic lesions, there are enzymes that synthesize estrogenfrom androgenic precursors as well as for *de novo* synthesis from cholesterol (Cornillie et al., 1990; Nap et al., 2004; Attar and Bulun, 2006; Kamergorodsky et al., 2009; Gibson et al., 2018; Mori et al., 2018; Mori et al., 2019). Conversely, estrogen-inactivating enzymes are clearly dysregulated in endometrial and/or endometriotic tissues evidencing a putative compensatory mechanism in view of the increased local estrogen concentrations (Piccinato et al., 2016b, 2017; 2018b).

Overall, two estrogen synthesizing pathways stand out: the aromatase (ARO)-dependent pathway and the steroid sulfatase (STS)-dependent pathway (Piccinato et al., 2018a). Briefly, ARO catalyzes the aromatization of androstenedione and testosterone to E1 or E2, respectively. STS mediates the local desulphonation of inactive circulating steroids, called estrogen sulfates, to their unconjugated, biologically active forms (Miller and Fraser, 2015; Foster, 2021). Interestingly, both pathways share a common final step through the action of hydroxysteroid 17-beta dehydrogenase (HSD17B1). This reducing enzyme belongs to the HSD17B family with high specificity for steroid substrates and high catalytic activity for the conversion of E1 to E2; therefore, HSD17B1 is an important regulator of intracellular steroid hormone concentration at the final stages of biosynthesis (Konings et al., 2018; Foster et al., 2019).

Although no clear association between lesion sites and enzymes function has been described in endometriosis, different types of lesions, as well as different localizations, seem to directly determine the dynamics of the disease (Taylor et al., 2021). Considering that endometriosis is an estrogen-dependent disease, the expression regulation of estrogen-synthesizing enzymes may have an even more pre-eminent impact on endometriosis dynamics. In this context, although sometimes with contradicting results, some studies have focused on site-specific regulation of aromatase. A couple of studies have described ovarian lesions/endometriomas (OMA) express high levels of aromatase gene (*CYP19A1*) and protein (ARO) (Heilier et al., 2006; Szaflik et al., 2020), whereas deep infiltrating endometriotic lesions (DIE) either fail to express ARO or show reduced expression (Bulun et al., 2004). Another example of site-specific regulation occurs with STS, which is highly expressed in DIE and OMA (Šmuc et al., 2007; Šmuc et al., 2009; Piccinato et al., 2016b) but is minimally present or even absent (Colette et al., 2009) in the eutopic endometrium of endometriosis-affected women (Dassen et al., 2007). The unbalanced redox metabolism of enzymes belonging to the HSD17B1 family is the putative cause of the higher E2 synthesis that has been detected in ectopic and eutopic endometrium. Expression of reducing type- *HSD17B1* mRNA was significantly higher in the ectopic tissue (superficial peritoneal endometriosis: SUP, DIE, and OMA), whereas no difference was seen in the expression of *HSD17B1*-oxidizing types 2 and 4 (Šmuc et al., 2007; Bulun et al., 2010; Delvoux et al., 2014). Altogether, these observations strengthen the idea that the production of estrogen, which takes place through complex enzymatic pathways, is site-specific.

There is no consensus among published studies regarding the expression of estradiol-synthesizing enzymes in endometriotic lesions. Furthermore, there are no published studies simultaneously investigating the expression of these enzymes, ARO, STS, and HSD17B1, in endometriotic tissue samples obtained from a large cohort of patients. The present study explores previous significant reports that demonstrate high and constant levels of estrogens (estradiol and estrone) in different endometriotic tissues (Huhtinen et al., 2012). We, thus, hypothesize that the observed site-specific variations in estrogen concentrations are a result of differential expression regulation of estrogen-synthesizing enzymes, namely, ARO and STS, and also HSD17B1.

Although intracrine estrogen concentration depends on the complex interaction of synthesizing and metabolizing enzymes, our aim was to evaluate gene and protein expression of the synthesizing part (ARO, STS, and HSD17B1) in different types of endometriotic lesions (SUP, OMA, and DIE) as well as in samples of eutopic endometrium (EE) obtained from the same patients with endometriosis. Patients without endometriosis were donors of nonendometriosis eutopic endometrial tissue, here called the control group. In addition, we investigated by laser-capture microdissection and immunohistochemistry the cellular expression of the chosen enzymes.

## Materials and Methods

### Patients and Samples

One hundred and ten women (94 with endometriosis and 16 without endometriosis: control group) were screened at the Assisted Reproduction Centre of the tertiary hospital of Faculdade de Medicina de Ribeirão Preto (Universidade de São Paulo, FMRP-USP), at the São Paulo Endometriosis Centre and Hospital Israelita Albert Einstein (HIAE), between the years 2011–2019. Biopsies of eutopic endometrium (control = control eutopic endometrium without endometriosis and EE = eutopic endometrium of a patient with endometriosis), superficial peritoneal endometriosis (SUP), deeply infiltrating endometriosis (DIE) (rectum, rectosigmoid, retrocervical, vesical, and uterosacral ligament lesions), and ovarian endometrioma (OMA) together with 5 ml of peripheral blood were collected in surgery.

**FIGURE 1:**
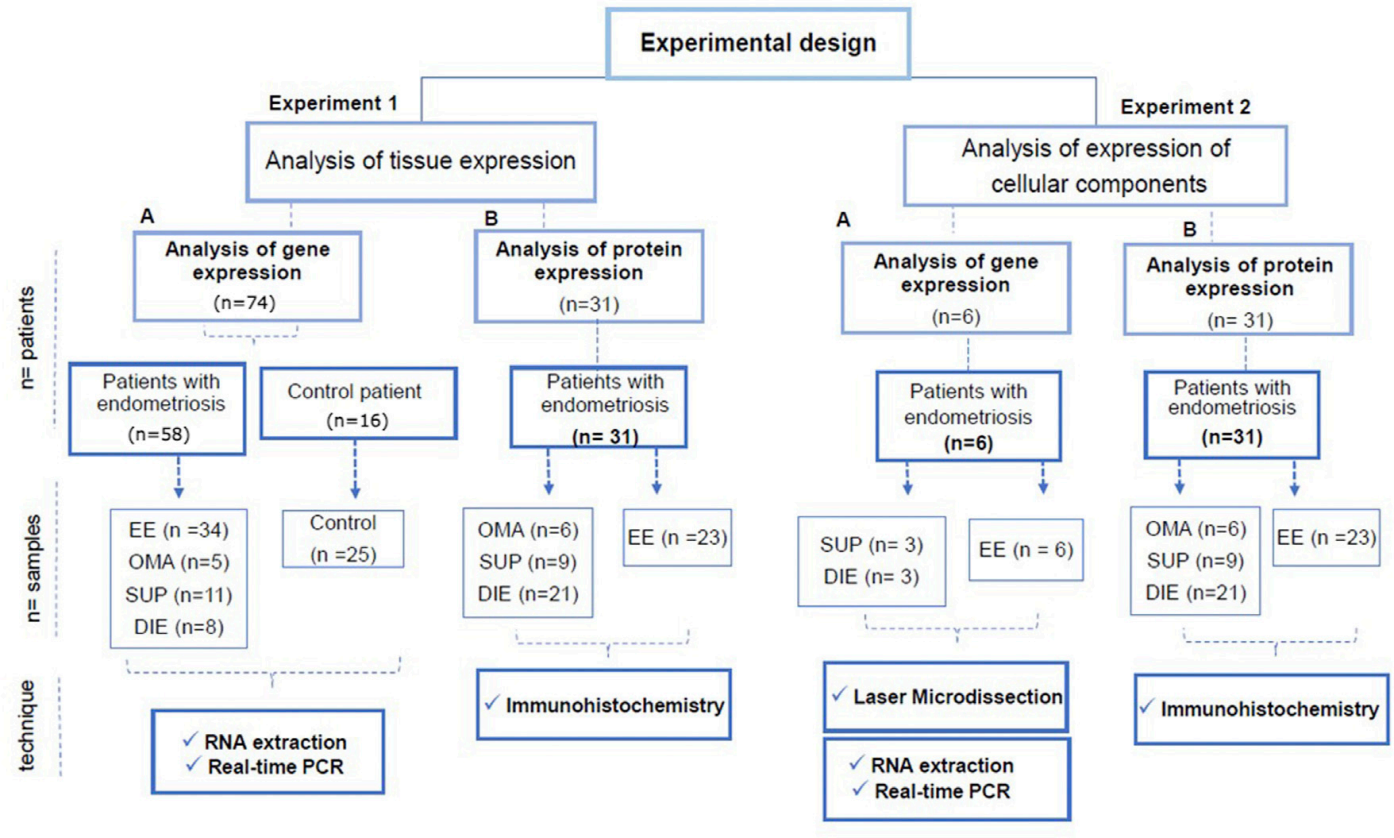
Experimental design of the study. The study was carried out in two types of experiments: Experiment type 1: analysis of gene and protein expression of the estrogen synthesizing enzymes in tissue samples. Experiment type 2: analysis of gene expression of estrogen synthesizing enzymes in microdissected areas containing glandular epithelium or stroma, and the protein expression analysis done by IHC in whole tissue sections. In experiments 1-A and 2-A, the samples were evaluated for gene expression of *ARO, STS*, and *HSD17B1* by RT-PCR. In experiments 1-B and 2-B, the samples were evaluated for protein expression of the enzymes (*ARO, STS*, and *HSD17B1*) by immunohistochemistry (IHC). In experiment 1-A, samples from 83 (endometriosis and control) were analyzed. Fifty-eight endometriosis patients provided 34 samples of eutopic endometrium, 5 ovarian lesions, 11 superficial peritoneal lesions, and 8 deep lesions. Samples from 16 control patients provided 25 biopsies of the endometrium. Some control patients provided samples from both menstrual cycle phases. In experiment 1-B, samples from six endometriosis patients were used, three of which were from superficial peritoneal lesions, three were from deep lesions, and six samples were from eutopic endometrium. In experiment 2-B, 31 patients provided 6 samples of ovarian lesions, 9 samples of peritoneal superficial lesions, 21 samples of deep lesions, and 23 samples of eutopic endometrium. *Abbreviations:* EE, eutopic endometrium of a patient with endometriosis; control, control endometrium; OMA, ovary endometriotic lesion; SUP, superficial peritoneal endometriotic lesion; DIE, deep endometriotic lesion.

The majority of patients enrolled for gene expression assessment are an extension of our sample cohort previously described (Piccinato et al., 2016b; Piccinato et al., 2018b), which includes proliferative and secretory phase samples. Figure 1 depicts the control patients provided biopsies from both menstrual cycle phases; thus, out of 16 patients enrolled, 25 biopsies were collected (12 nonsecretory and 13 secretory). Endometriosis patients provided together 34 eutopic endometria (8 nonsecretory and 26 secretory), 5 endometrioma (2 nonsecretory and 3 secretory), 11 superficial lesions (1 nonsecretory and 10 secretory), and 8 deep infiltrating lesions (3 nonsecretory and 5 secretory). For protein expression evaluation, 34 endometriosis patients (19 nonsecretory and 15 secretory) provided 23 eutopic endometria, 6 endometriomas, 9 superficial lesions, and 21 deep infiltrating lesions. No difference in age was observed among patients (average of 35.5 ± 5 years).

All samples were collected after a written, informed consent form was signed by all patients. The study was approved by the Committee on Human Research of the Hospital Albert Einstein (FR-30468, 12/16/2009; São Paulo, Brazil), which is affiliated to the Ethics Committee of the Brazilian Ministry of Health (CONEP), upon signing the informed consent form.

The diagnosis of endometriosis was made by visualizing the lesions during surgery, which was further confirmed by histopathological analysis at the Pathological Anatomy Laboratory of FMRP-USP or HIAE. Nonendometriosis patients had confirmed the absence of endometriotic foci by inspection during tubal ligation surgery.

Inclusion criteria for both groups were reproductive age, nonsmokers, non-menopause, and no use of any hormonal therapy for at least 3 months before collection. Exclusion criteria were the presence of other reproductive disorders such as polycystic ovary syndrome, myoma, or any tumors.

### Experimental Design

A diagram of the experimental design is shown in Figure 1. Basically, two types of experiments were performed. The gene and protein expression of estrogen synthesizing enzymes quantified by RT-PCR and immunohistochemistry (IHC) with the histometric analysis in extracted fragments from the lesion biopsies or, as a second approach, in the thereof microdissected or immunohistochemistry-stained endometrial glandular and stromal regions.

In all experiments, gene expression analyses were performed by RT-PCR and protein expression analyses were by immunohistochemistry. The patients’ menstrual cycle phase was considered in all protein assessments. The menstrual cycle phase was defined by the dosage of progesterone: secretory (progesterone >1 ng) and nonsecretory (progesterone <1 ng).

### Laser Capture Microdissection

The laser capture microdissection (LCM) technique was used to obtain separate samples of regions containing glandular cells or stromal cells. The preparation of histological slides (Membrane Slide NF 1.0 PEN D Zeiss, Munich, Germany) for LCM was performed in a cryostat (Leica CM 1860; Buffalo Grove, IL, United States), following the manufacturer’s instructions. The samples were embedded, with the aid of an Optimal Cutting Temperature (OCT) compound (Sakura Finetek, Torrance, CA, United States), and cut into 10 *μ*m sections. The slides were stained with Crystal Violet (Sigma Aldrich, MERK SA, Darmstadt, Germany) according to a protocol already tested by our group (Da Costa et al., 2019). The LCM was performed with the computerized system PALM RoboSoftWare 4.6 MicroBeam LSM 710 visualized with a 20x objective. Areas larger than 50 *μ*m^2^ were microdissected according to previous standardization of the LCM technique (Da Costa et al., 2019). The microdissected materials were collected in microdissection tubes (Sample AdhesiveCap 500 clear D Zeiss, Munich, Germany) and immediately frozen at −80°C.

Whenever needed, a pathologist validated the identification of the two areas, glandular and stromal, in the microdissected samples of EE and endometriotic lesions. Glandular and stromal areas from EE were, respectively, compared with glandular and stromal areas from endometrial lesions.

### RNA Extraction

The PicoPure RNA Isolation Kit (Life Technologies, MERK SA, Darmstadt, Germany) was used to extract RNA from whole biopsy samples, whereas for the microdissected samples, a specific kit for microdissected samples, RNAaqueous Micro Total RNA Isolation Kit (ThermoFischer Scientific, Waltham, MA, United States) was used. The extracted total RNA was quantified by spectrophotometry using a NanoDrop One Microvolume UV-Vis Spectrophotometer (ThermoFisher Scientific, Waltham, MA, United States) at an absorbance of 260 nm and frozen at −80°C.

### cDNA Synthesis and Real-Time Polymerase Chain Reaction (RT-PCR in Real Time)

Reverse transcription of RNA samples was performed using the SuperScript III First-Strand Synthesis SuperMix Kit (Invitrogen, ThermoFisher Scientific, Waltham, MA, United States) with oligo-dT primers. Some RNA samples needed dilution in DNase/RNase free ultrapure water and others were concentrated using a vacuum concentrator (Refrigerator Centrivap Vaccum Concentrators- Labconco, Kansas City, MO, United States). The final reaction volume was 14 μl per sample. The samples were placed in a thermocycler (Master Cycler–Nexus, Eppendorf Sigma Aldrich, MERK SA, Darmstadt, Germany) at 50°C for 50 min.

The sequences of genes analyzed and synthesized by Invitrogen Brazil were CYP19A1 forward = 5-CAC​AGA​AGA​GAA​ACT​GGA​AGA​A-3 and reverse = 5- TCC​AAT​ATG​CAC​TGG​TTC​AC-3, STS forward = 5-GGA​GTG​AGA​AGG​GCA​TGG​TA-3 and reverse = 5-CTC​CAG​CAG​CCT​CTT​TAT​GG-3, HSD17β1 forward = 5-TCG​CGT​TAG​CCA​GTT​TTA​CC-3 and reverse = 5-TCG​CGT​TAG​CCA​GTT​TTA​CC-3, and GAPDH (housekeeping) forward = 5-GAA​GGT​GAA​GGT​CGG​AGT​CA-3 and reverse = 5-TGA​GGT​CAA​TGA​AGG​GGT​CA-3.

Real-time amplification was performed according to the SYBRGreen Master Mix assay protocol (Thermo Fisher Scientific, Waltham, MA, United States). Briefly, 1.5 *μ*l of cDNA was added to 13.5 *μ*l of the mix, totaling the final volume of 15 *μ*l of reaction per well of the plate. The specificity of the generated product was confirmed by analyzing the dissociation curve of the primers (melting curve) of the formed products as well as by electrophoretic running of the material amplified on a 2.5% horizontal agarose gel. The calculations of the relative gene expression of the samples were made using the ΔΔCt method with the housekeeping gene *GAPDH* as normalizer. The normalizer was determined based on the coefficient of variation (CV) obtained in similar datasets, being the lowest when compared to other candidate housekeeping genes (Piccinato et al., 2016b; Piccinato et al., 2018b).

To calculate Δ^−Ct^ as well as to control intra-assay variation, placental cDNA was used as a reference sample for the analysis of gene expression of the biopsy (whole tissue) (Piccinato et al., 2016a). For the analysis of gene expression of cellular components, a pool of samples of 2 *μ*l of cDNA from tissue samples (non-microdissected) was used as a reference sample.

### Immunohistochemistry and Histometric Analysis

Paraffin-embedded blocks were used. 3 *μ*m thick sections spaced by 9 *μ*m were obtained (microtome: Leica RM2125 RTS, Buffalo Grove, IL, United States).

Immunohistochemistry was performed using the BenchMarck ULTRA IHC/ISH labeling platform from Ventana Medical System Inc. (Tucson, Arizona, United States) using the ultraView Universal DAB Detection kit, following the manufacturer’s guidelines. In brief, slides were blocked with hydrogen peroxide and incubated at 35°C with 100 μL of primary antibody 1:200 anti-ARO (ab18995, Abcam, Cambridge, United Kingdom) and 1:200 anti-HSD17B1 antibodies (ab51045, Abcam, Cambridge, United Kingdom) for 1 h and antiSTS 1:100 (ab62219, Abcam, Cambridge, United Kingdom) for 2 h. The samples were incubated with polymer, chromogen, and stained with hematoxylin. At the end of the procedure, the slides were washed in a solution with Tween 20 detergent (Sigma-Aldrich, MERK SA, Darmstadt, Germany), dehydrated to 100% alcohol, and mounted with coverslips and mounting medium (Dako, Mounting Medium).

The stained slides were analyzed using the IX51 Microscope and Olympus cellSens Dimension 1.16 software (Shinjuku, Tokyo, Japan). To evaluate the protein expression of the whole tissue samples, three different fields from each section were photomicrographed at ×10 magnification, resulting in nine images per sample. To evaluate protein expression, two different fields of each section were photomicrographed (at ×20), resulting in six photomicrographs per sample. Similarly, the same photomicrographs were used to determine enzyme staining at different cellular components: glandular or stromal areas. Protein expression values were determined from the histometric analysis of the percentage of the stained area for each of the proteins (ARO, STS, and HSD17B1) (Bartels et al., 1995).

### Statistical Analysis

The variables of interest in the present study, gene and protein expression of ARO, STS, and HSD17B1 and their ratios, were continuous, nonnormal, and asymmetrically distributed, being analyzed by generalized models with Weibull distribution or generalized estimating equation models with Tweedie distribution, in order to compare groups (EE, SUP, OMA, and DIE) and menstrual cycle phase (secretory and nonsecretory). The ratio between enzymes (ARO:STS, ARO:HSD17B1, and STS:HSD17B1) was a mathematical strategy to evaluate the relative expression level between enzymes per sample. The models adjusted considered the interaction effect between the groups and menstrual cycle phase, the dependence between the measurements of the same patient in different tissues (e.g., EE and lesions), and the multiple comparisons were corrected by the sequential Bonferroni method. The results are presented as estimated means and 95% confidence intervals (CI). Spearman’s correlation was applied to evaluate the relationship among variables. Analyses were performed using the SPSS program, R, and GAMLSS package, considering a *p value* of less than 0.05 statistically significant.

## Results

### Higher *CYP19A1* Expression Occurs in Endometriosis Tissue, and Higher *ARO* Occurs in Eutopic Endometrium as Compared to Lesions

**FIGURE 2:**
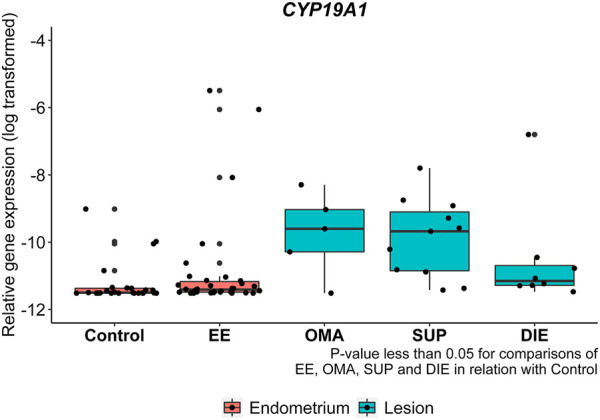
*CYP19A1* mRNA expression from endometriotic samples. Gene expression levels of *CYP19A1* were analyzed by real-time polymerase chain reaction in eutopic endometrium from control patients (control), eutopic endometrium from endometriosis patients (EE), and in ovarian (OMA), superficial (SUP), and deep-infiltrating (DIE) lesions. Relative gene expression levels were normalized by *GAPDH*. Bars represent the log_10_ mean fold change of the normalized gene expression relative to a reference sample and 95% CI. Statistical differences between groups are indicated by *p < 0.005.

We compared the gene expression between endometriosis tissues and control and protein expression among endometriotic lesions and EE. Notably, expression levels of *CYP19A1* were significantly higher in all types of endometriotic lesions OMA (12 times, *p* = 0.0390), SUP (31 times, *p =* 0.0171), and DIE (16 times, *p* = 0.0446), as well as in the EE (4 times, *p* = 0.039), compared to control endometrium (Figure 2).

**FIGURE 3:**
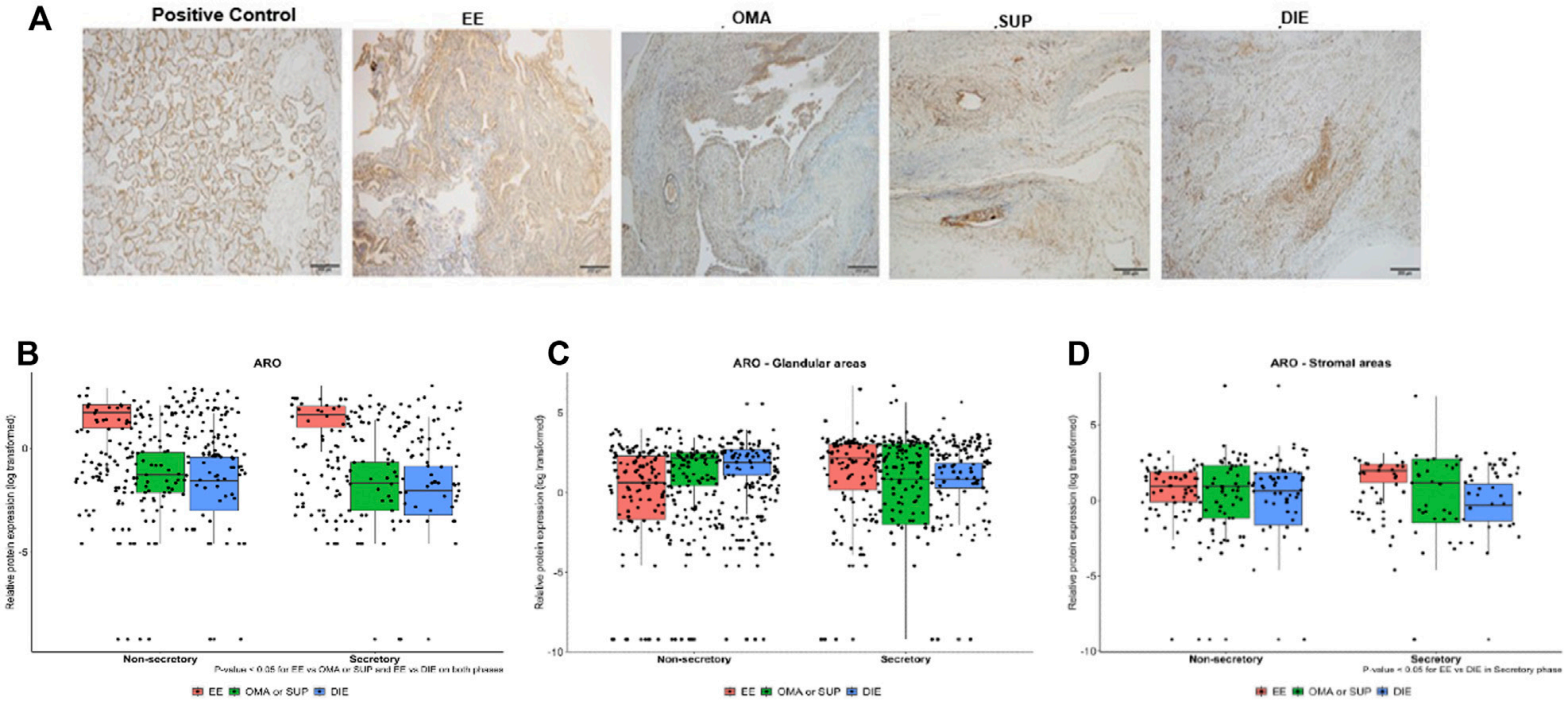
Expression of aromatase in endometriotic lesions from different locations obtained in the secretory and nonsecretory phases. **(A)** Representative photomicrographs from IHC aromatase-stained sections of EE, OMA, SUP, and DIE endometriotic lesions and from the placenta (positive control). Aromatase protein expression levels quantified by IHC in biopsy sections obtained at nonsecretory and secretory phases of the menstrual cycle: **(B)** whole sections, **(C)** glandular areas, and **(D)** stromal areas. Bars represent the log_10_ mean fold change of the relative protein expression, accompanied by 95% CI. Statistical differences between groups are indicated by *p < 0.005. EE: eutopic-endometrium (*n* = 23); OMA: ovarian (*n* = 6); SUP: superficial (*n* = 9); and DIE: deep-infiltrating (*n* = 21) lesions from endometriosis patients. Group OMA/SUP: the analysis by IHC showed similar values and distribution for OMA and SUP lesions, and their data were joined into one group OMA/SUP (*n* = 15) for statistical analysis purposes.

When whole sections were analyzed by IHC (Figure 3A) for ARO protein expression, higher expression of this enzyme was detected in EE compared to OMA/SUP (*p* < 0.003) and DIE (*p* < 0.001) at both menstrual phases (Figure 3B).

Next, we looked at ARO protein expression in the stromal and glandular regions of EE, OMA/SUP, and DIE biopsies (Figure 3C and 3 days). Both regions, stromal and glandular, of the three types of biopsy locations, expressed ARO at similar levels, at both menstrual phases (Figure 3C and 3 days). The only significant difference in ARO expression was detected in the stromal area of DIE (circa 10-fold higher) vs. EE at the secretory phase (Figure 3D).

### Gene and Protein Expression of *STS* and *HSD17B1* Are Higher in Endometriotic Lesions Than in EE

**FIGURE 4:**
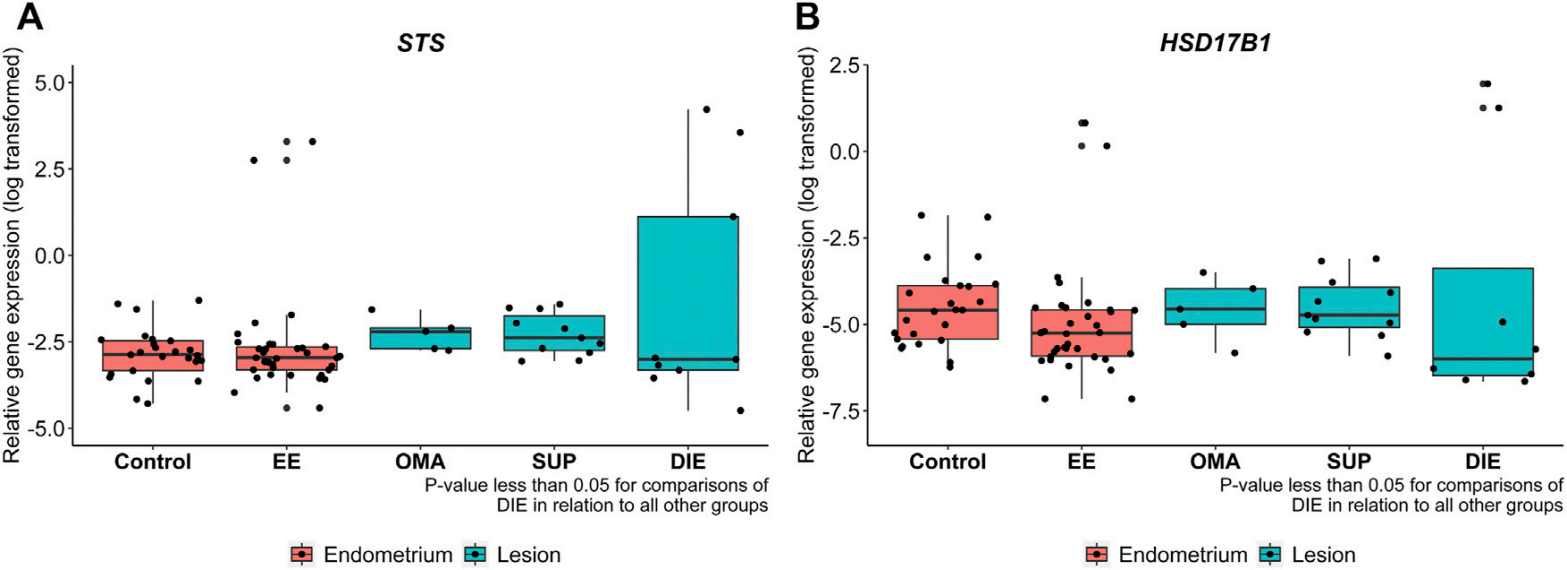
*STS* **(A)** and *HSD17B1* **(B)** mRNA expression in endometriotic and nonendometriosis endometrium control samples. Gene expression levels of STS and HSD17B1 were analyzed by qRT-PCR in endometrium from control nonendometriosis patients (control) and eutopic endometrium from endometriosis patients (EE), ovarian (OMA), superficial (SUP), and deep-infiltrating (DIE) lesions. Relative gene expression levels were normalized by GAPDH. Bars represent the log 10 mean fold change of the normalized gene expression relative to a reference sample, and 95% CI. Statistical differences between groups are indicated by *p < 0.005.

**FIGURE 5:**
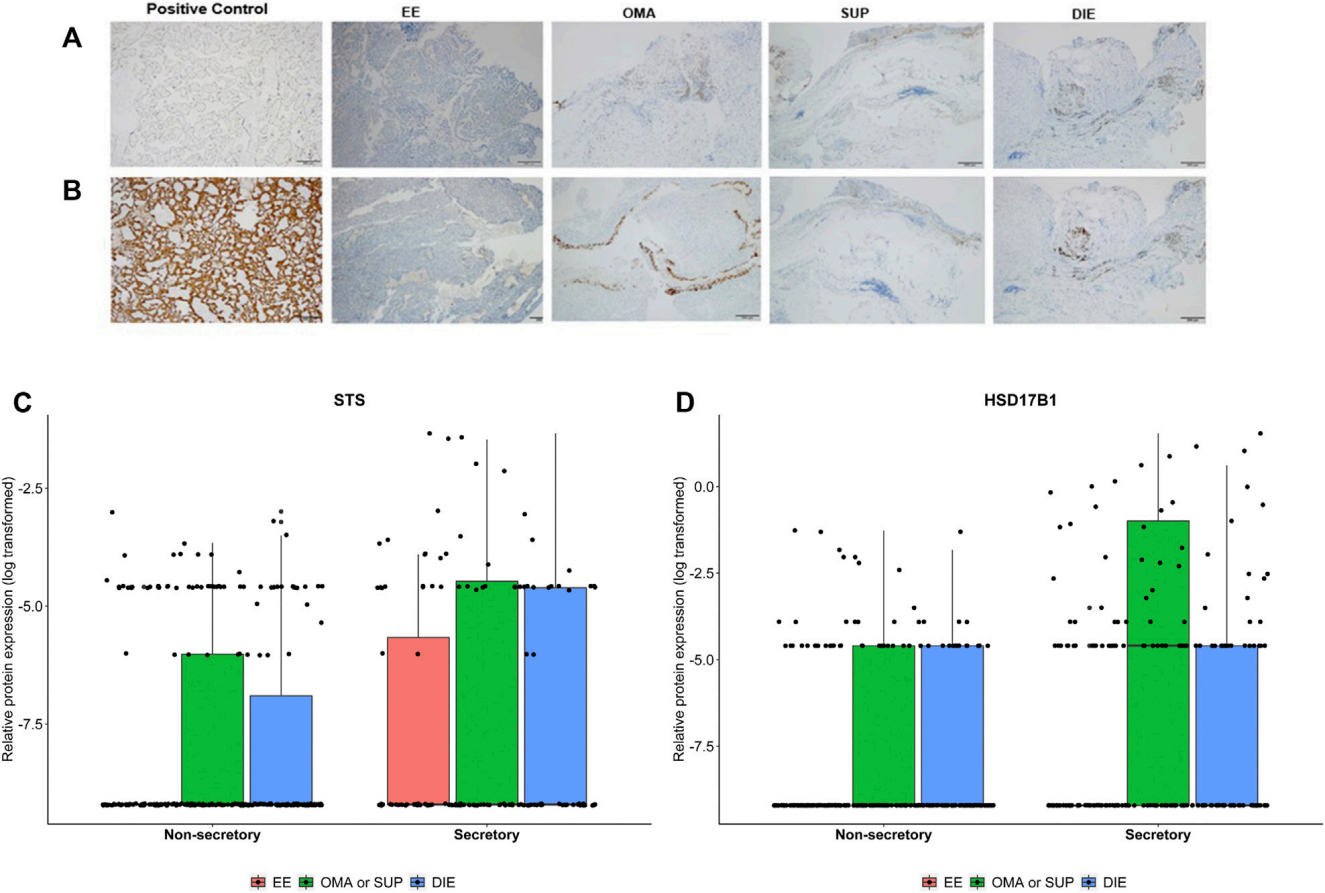
Representative images from *STS* **(A)** and *HSD17B1* **(B)** protein expression in endometriotic samples and positive control (placenta) analyzed by immunohistochemistry. Protein tissue expression levels of *STS* **(C)** and *HSD17B1* **(D)** in eutopic endometrium from endometriosis patients (EE), ovarian (OMA), superficial (SUP), and deep-infiltrating (DIE) lesions. Samples were divided into a nonsecretory and secretory phase of the menstrual cycle. Bars represent the log_10_ mean fold change of the relative protein expression, accompanied by the 95% CI.

Figure 4 depicts RT-qPCR results regarding tissue *STS* and *HSD17B1* gene expression. Only DIE had significantly higher expression levels of *STS* and *HSD17B1* in comparison to all other collected samples including control (*p* = 0.0003 and *p* = 0.0163, respectively), EE (*p* = 0.018 and *p* = 0.0035, respectively), and other endometriotic sites (OMA: *p* = 0.0223 and *p* = 0.0201, respectively, and SUP: *p* = 0.0004 and *p* = 0.0035, respectively). Apart from DIE, all other groups showed similar levels of *STS* or *HSD17B1* mRNA expression. Tissue protein analyses by IHC done on whole tissue sections for either STS (Figure 5A) or HSD17B1 (Figure 5B) showed no statistical differences among groups (Figure 5C,D).

**FIGURE 6:**
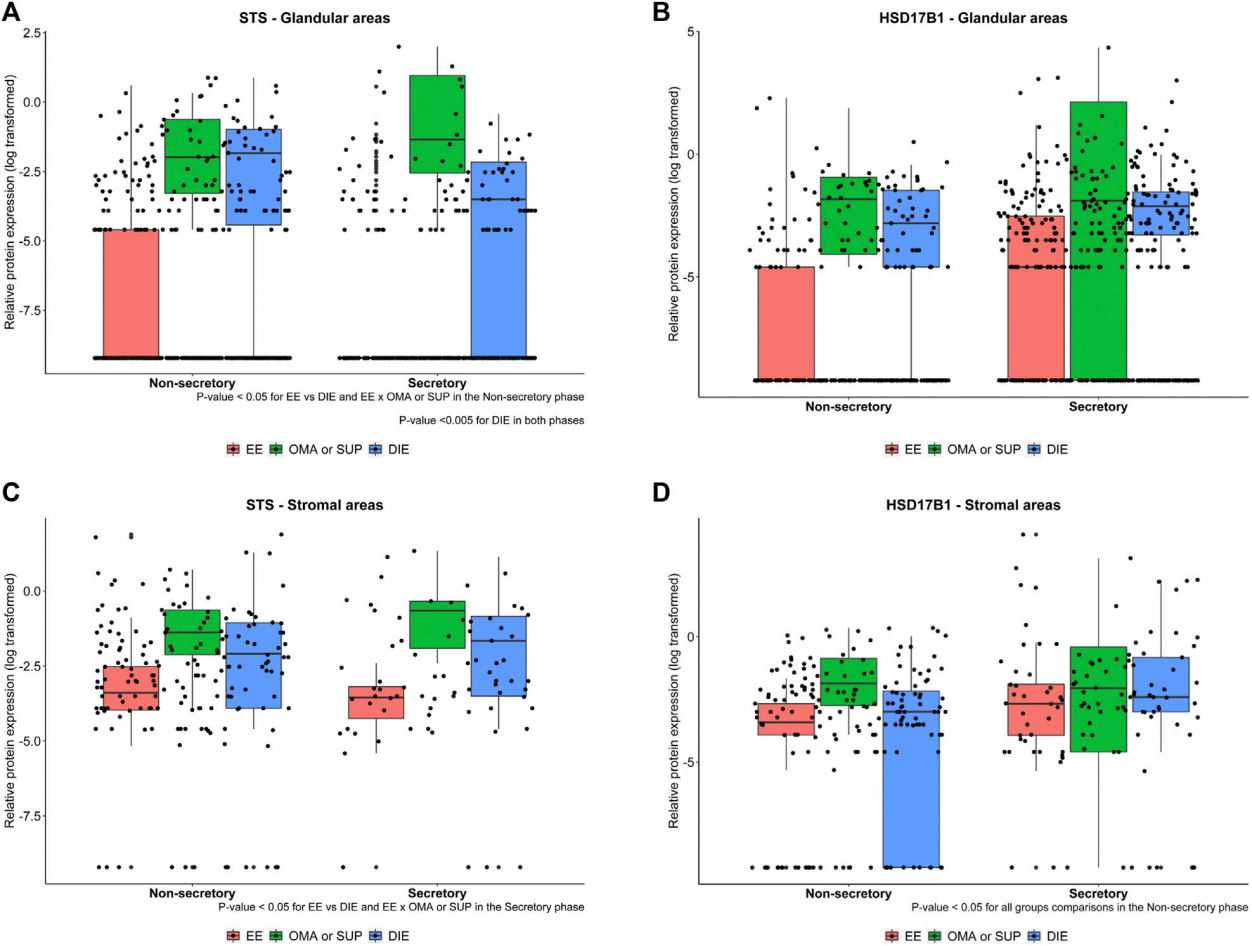
Representative images from STS and HSD17B1 protein expression in glandular **(A, B)** and stromal areas **(C, D)** from endometriotic samples. Protein expression levels of STS and HSD17B1 were analyzed by immunohistochemistry in eutopic endometrium from endometriosis patients (EE), ovarian (OMA), superficial (SUP), and deep-infiltrating (DIE) lesions. Samples were divided into nonsecretory and secretory phase of the menstrual cycle. Bars represent the log_10_ mean fold change of the relative protein expression, accompanied by 95% CI. Statistical differences between groups are indicated by *p < 0.005.

However, when areas of sections were analyzed for STS protein expression, OMA/SUP and DIE groups had (Supplementary Figure S1), in the glandular areas, higher values than in eutopic endometrium from patients (EE) at both menstrual cycle phases (Figures 6A,B). In the stromal areas, again OMA/SUP (*p* = 0.018) and DIE (*p* = 0.012) groups had *circa* two-fold higher values for STS expression than EE. In contrast, HSD17B1 was present in the glandular and stromal areas, but differences between groups of *circa* two-fold were detected only at nonsecretory-phase samples for OMA/SUP (*p* < 0.001) or DIE (*p* = 0.012) vs. EE (Figures 6A,B).

### *STS* Is the Most Expressed Gene in Endometriosis, but ARO Is the Most Expressed Enzyme

Ratio analyses were employed to compare groups regarding enzyme relationships. The gene and protein ratios between estrogen-synthesizing enzymes were chosen to evaluate the regulation of ARO, STS, and HSD17B1. For these analyses, the interaction effect between the group and the cycle phase classification was taken into consideration. As shown, STS had the highest values for gene expression, and ARO had the highest ones for protein comparisons (Tables 1,2). The gene expression ratios, *CYP19A1:STS* and *CYP19A1:HSD17B1*, were significantly higher in endometriotic lesions compared to EE and control*.* These results indicate that *CYP19A1* was lower than STS in OMA (*p* = 0.022) e SUP (*p* = 0.004) compared to control, and in SUP (*p* = 0.012) compared to EE. In addition, *CYP19A1* was lower than HSD17B1 in OMA (*p* = 0.005), SUP (*p* = 0.0005), and DIE (*p* = 0.039) in relation to control, and lower in SUP (*p* = 0.049) when compared to EE. There was also a difference in *CYP19A1:HSD17B1* ratio between EE and control (*p* = 0.011) (Table 1).

**TABLE 1:** Gene expression ratio among *CYP19A1*, *STS*, and *HSD17B1* in biopsies from endometriotic lesions and from eutopic endometrium of non-endometriosis patients (control).

	Ratio *CYP19A1:STS* (×10^−5^)	Ratio *CYP19A1:HSD17B1* (×10^−5^)	Ratio *HSD17B1:STS* (×10^−8^)
Control	2.4 (0.6; 10.5)	9.9 (2.3; 41.7)	39.4 (21.6; 71.8)
EE	6.7 (2.9; 15.5)	76.1 (25.9; 223.5)^	14.7 (10.9; 19.9)
OMA	21.4 (0.9; 526.5)*	146.5 (3.2; 6682.5)^^	15.3 (7.5; 31.6)
SUP	65.2 (22.6; 188.1)*#	584.2 (210.3; 1623.1)^^**	15.9 (10.5; 24.0)
DIE	11.0 1 (5.3; 23.0)	155.7 (49.8; 487.3)^	10.4 (7.0; 15.6)

Results are expressed as mean and 95% CI. *p < 0.01 vs. control. ^#^ *p* < 0.01 vs. EE. ^#^ *p* < 0.05 vs. EE within cycle phase. ^ *p* < 0.05 vs. control. ^^ *p* < 0.001 vs. control. **p < 0.05 vs. EE.

**TABLE 2:** Protein expression ratio among *ARO*, *STS*, and *HSD17B1* in biopsies from endometriotic lesions and from eutopic endometrium of nonendometriosis patients (control).

	Cycle phase	Ratio ARO:STS (×10^−1^)	Ratio ARO:HSD17B1 (×10^−1^)	Ratio STS:HSD17B1
EE	Nonsecretory	59.8 (42.1; 84.8)	57.3 (25.2; 130.6)	0.38 (0.05; 0.89)
	Secretory	57.8 (28.6; 117.0)	66.7 (31.8; 139.6)	0.43 (0.1; 0.84)
SUP-OMA	Nonsecretory	11.9 (6.0; 23.8)*^	7.1 (2.7; 18.7)	0.31 (0.13; 0.59)
	Secretory	1.8 (1.0; 3.1) #^	8.2 (2.7; 25.1)	0.36 (0.11; 0.72)
DIE	Nonsecretory	3.3 (1.0; 11.3)*	3.4 (1.6; 7.2)	0.31 (0.11; 0.63)
	Secretory	1.3 (0.7; 2.4)#	3.9 (2.1; 7.4)**	0.36 (0.15; 0.65)

Results are expressed as mean (95% CI). *p < 0.01 vs. EE within cycle phase. ^#^ *p* < 0.05 vs. EE within cycle phase. ^ *p* < 0.05 nonsecretory vs. secretory phase. **p < 0.05 vs. EE within cycle phase.

At the protein level, comparisons of the ARO:STS ratio among all groups highlighted that the ARO expression was consistently greater than the STS expression (Table 2). The ARO expression prevailed over the STS expression in EE, in both cycle phases, with higher ratio in EE as compared to OMA:SUP lesions (*p* < 0.001 in the nonsecretory phase; *p* = 0.017 in the secretory phase) and DIE (*p* < 0.001 in the nonsecretory phase; *p* = 0.017 in the secretory phase). In all compared groups, the ARO: HSD17B1 ratios indicate that ARO was greater than the expression of HSD17B1 (Table 2). The ARO: HSD17B1 ratio was higher in EE when compared to DIE (*p* = 0.032) in the secretory phase but not among the other experimental groups. No other differences were detected among endometriotic sites. Notably, the STS: HSD17B1 ratio did not vary among all compared groups (*p* > 0.05).

The Spearman correlation analysis was employed to evaluate the relationship among gene and protein expression of enzymes regardless of groups. The results of mRNA expression showed weak and positive correlation between *CYP19A1* and *STS* (ρ = 0.052, *p =* 0.001) and between *CYP19A1* and *HSD17B1* (ρ = 0.033, *p* = 0.0005). Furthermore, there was a stronger and positive correlation between *STS* and *HSD17B1* (ρ = 0.402, *p* < 0.001). Correlation among protein levels demonstrated weak and negative correlation between ARO and HSD17B1 (ρ = −0.152*, p* = 0.001) and weak and positive correlation between STS and HSD17B1 (ρ = 0.133, *p* = 0.009). ARO e STS demonstrated weak negative correlation with no statistical difference (ρ = −0.051 e *p* = 0.310).

### No Detectable Differences in the Expression of *CYP19A1*, *STS*, and *HSD17B1* mRNA Were Observed at Cellular Levels

*CYP19A1* was detected in 20.8% of samples, while *STS* was identified in 83% of stromal and 66% of epithelial cells. *HSD17B1* expression was present in almost all samples: 91.6% stromal and 91.6% epithelial (Supplementary Figure S2).

## Discussion

Our hypothesis in this study was that previously reported estrogen levels detected in endometriosis sites (Huhtinen et al., 2012) are different in function of the distinct expression of estrogen-synthesizing enzymes in each lesion site. Our data show that the relative expression of *ARO*, *STS*, and *HSD17B1* vary in endometriotic tissue obtained from deep infiltrating lesions (DIE), superficial (SUP), ovarian (OMA), and eutopic endometrium from endometriosis patients (EE) lend support to the hypothesis. The expression of those enzymes in endometriosis was also compared to endometrium samples of nonendometriosis patients (control). It should be mentioned that we analyzed material from a large number of patients taking into account the patients’ menstrual cycle phases.

The gene encoding *ARO*, *CYP19A1*, was detected in all endometriosis tissue at levels all greater than found in control; *STS* and *HSD17B1* presented consistently higher mRNA expression levels in DIE. In addition, analysis of gene expression levels applied to the dataset indicated that *STS* and *HSD17B11* genes were the most closely regulated. Overall, for protein expression, EE presented higher levels of ARO and endometriotic lesions had superior levels of *STS* and *HSD17B1*. Particularly, unique patterns of mRNA expression levels of the *STS* and *HSD17B1* were described in DIE when compared to OMA and SUP lesion sites; EE gene and protein expression regulations of *ARO*, *STS*, and *HSD17B1* were distinct from those of the endometriotic sites. Taken together, the findings of different levels of the estrogen-synthesizing enzymes in distinct lesion sites support the hypothesis that the local tissue microenvironment can influence and be influenced by the regulation of different estrogenic pathways. Ultimately, this determines the local availability of estrogen, previously reported in other studies (Huhtinen et al., 2012), which in turn directly affects the local growth of ectopic endometrium.

Gene expression (mRNA) and tissue protein expression (as measured in IHC stained sections) of *ARO*, *STS*, and *HSD17B1*, as well as their relative ratios, did not always match. *STS* mRNA was more expressed in relation to the others enzymes, in all sample groups analyzed, followed by *HSD17B1* and finally by *CYP19A1*. ARO protein expression was higher among groups, followed by *STS* and *HSD17B1*. We detected differences between *CYP19A1:STS* ratios, *CYP19A1:HSD17B1* (gene expression), *ARO*:*STS*, and *ARO:HSD17B1* (protein expression) in different sites of endometriosis. This would reinforce the idea that there is a regulation of enzymes in a site-specific manner, as the proportions are variable among sites. On the other hand, the lack of difference between gene and protein expression for the ratio *HSD17B1:STS*, in different endometriosis sites indicates that these enzymes are more closely regulated, as they show less expression variation. Supporting this observation, the Spearman correlation analysis of gene expression between *STS* and *HSD17B1* revealed a positive, moderate correlation between these same enzymes, not seen between them and *CYP19A1*. Correlation among protein levels was, in general, weak and, although significant, might not be clinically relevant.

It seems that endometriotic lesion sites’ estrogen synthesis is more closely regulated by the enzymes *STS* and *HSD17B1*, whereas in the EE, *ARO* is the dominant regulator. Although STS expressed higher mRNA but not protein in comparison with the other enzymes, the stromal areas of OMA/SUP and DIE lesions showed STS elevated levels of protein. Conversely, ARO was significantly more expressed at the protein level, and it was prominent in stromal areas of EE.

The existence of tissues similar to the endometrium in sites other than the uterine cavity implies that there are local and specific mechanisms that support the survival and growth of the ectopic tissue in an otherwise alien environment. Comparative to the other sites, DIE presented the most distinct and elevated *STS* and *HSD17B1* mRNA expression levels. This suggests that a unique hormonal environment developed in this lesion type favoring high estrogen-synthesis enzymes. Indeed, estradiol measurements by HPLC-MSMS showed its high and persistent concentrations in endometriotic lesions, including deep lesions (Huhtinen et al., 2012). Because vascularization is limited in the DIE (Liu et al., 2008) and the antagonizing enzyme, sulfotransferase is upregulated (Piccinato et al., 2016a), and it seems reasonable to propose that regulatory mechanisms might exist to maintain estradiol levels and disease progression despite poor blood irrigation. In OMA and largely in SUP lesions, blood supply is more pronounced favoring the mRNA expression of *CYP19A1*, allowing the use of the precursor’s testosterone/androstenedione from circulation to synthesize estradiol.

Either higher levels, or no difference, or absence of *CYP19A1* mRNA was previously described in endometriotic lesions in comparison with the endometrium of disease-free, women-control, or of endometriosis patients, EE (Noble et al., 1997; Matsuzaki et al., 2006; Dassen et al., 2007; Colette et al., 2009; Mori et al., 2018). Despite having verified that there is *CYP19A1* expression in control endometrium (although at lower levels compared to EE), this study corroborates previous findings showing increased gene and protein expression of ARO in EE (Hudelist et al., 2007; Aghajanova et al., 2009).

The enzymes STS and HSD17B1 have been considered important in the pathogenesis of endometriosis, as indicated by the use of their inhibitors in the treatment of endometriosis (Purohit et al., 2008; Salah et al., 2017; Maltais et al., 2018; Barra et al., 2019). Our data evidenced a parallel regulation between them suggesting a local combined relationship. Thus, *STS* and *HSD17B1* together can play an important role in estrogen production by desulfating sulfated steroidogenic compounds (DHEAS, E2S, and E1S) into active compounds that reach endometriotic tissues in the bloodstream (Purohit et al., 2008). Expression of STS (gene and protein), being greater than of HSD17B1, may provide more E1 or E2, but the final balance between these two estrogens depends on HSD17B1 regulating the formation of the more active estradiol, namely, E2.

In consonance with other authors (Konings et al., 2018), we found in a larger cohort of patients that the expression of *STS* and *HSD17B1* genes was much higher in DIE compared to all other endometriotic lesion types and EE or endometrium from nonaffected women. Increased *STS* mRNA expression has also been demonstrated in OMA, whereas *HSD17B1* expression was higher in all types of endometriotic lesions when compared to control (Šmuc et al., 2007) or EE (Mori et al., 2015). In fact, only low levels of the HSD17B1 gene and protein (Dassen et al., 2007) or total absence of this enzyme (Utsunomiya et al., 2001) were reported in the endometrium of nonaffected women. Colette et al. (2013) described similar levels of 1 HSD17B1 gene and protein expressions among different types of lesions.

The elevated expression of *STS* and *HSD17B1* genes in DIE lesions in comparison to other lesion types was not paralleled by the corresponding protein expression of these enzymes. Although staining by IHC was present for both enzymes, there were no significant quantitative differences between the analyzed groups (EE, SUP, OMA, and DIE). Other studies have also failed to find significant differences in STS protein expression among different endometriotic lesions and EE (Dassen et al., 2007).

The lack of association between the results of gene and protein expressions of the analyzed estradiol-producing enzymes can be explained by posttranscriptional regulatory events, which can prevent the formation of functional proteins from the mRNA transcript. Estrogens are consistently present in endometriotic tissue, regardless of the menstrual cycle phases (Huhtinen et al., 2012). Interestingly, the stability of several mRNAs is regulated by estrogens (Ing, 2005). Several microRNAs (miRNA) control gene expression at the posttranscriptional level and can be induced or inhibited by estrogen (Klinge, 2012; Kolanska et al., 2021). In addition, E2 canonical signaling pathways mainly mediated by nuclear estrogen receptors can perform as transcription factors to stabilize or destabilize mRNAs *via* miRNAs modifying gene expression (Kim et al., 2021). These controls promote the expression of genes that are critical to either strengthen or diminish the effects of steroid hormones that depend on the feedback of the same enzymes they regulate. Furthermore, estrogen-regulated expression of miRNA is both cell- and tissue-specific (Cohen et al., 2008).

Regarding the participation of the glandular versus stromal areas of endometriotic lesions in estrogen synthesis, we looked at the expression of *ARO, STS*, and *HSD17B1* in the various types of endometriotic lesions and of eutopic endometrium, EE. Molecular and histophysiological differences between stromal and glandular cells from EE or endometriotic lesions have already been described such as alterations in prosurvival enzymes, cytoskeletal proteins, proteasomes, and cell repair mechanisms (Bastos Wolff et al., 2012). It was suggested that endometrial stromal cells responsive to progesterone could induce HSD17B2 (i.e., inactivation of E2) in epithelial cells (Yang et al., 2001) via paracrine factors. However, primary cultures of OMA stromal cells stimulated with progesterone resulted in the suppression of *HSD17B2* expression in epithelial Ishikawa cells (Cheng et al., 2007).

We found higher protein expression of HSD17B1 in the stromal cell regions from endometriotic lesions. This result lends support to the idea that the regulation of estrogen secretion in endometriotic lesions is cell-specific. A pilot study detected, by immunofluorescence, the presence of stromal cells (but not epithelial cells) in the bloodstream of women with endometriosis, suggesting that endometrial stromal cells may migrate from the EE to distant sites via the bloodstream (Lin et al., 1995). It may also suggest that stromal cells could participate in the maintenance of endometriotic lesions. For the STS pathway, both stromal and epithelial cells seem to act at maintaining high levels of estrogen-synthesizing enzymes, as observed in our results. It appears that stromal cells are more susceptible to deficient steroid hormonal secretion.

Because steroid hormones regulate the expression of some estrogen metabolizing and synthesizing enzymes, which could be present at different levels throughout the menstrual cycle, the experimental design of our study included samples from progesterone-secretory and nonsecretory phases. Although the menstrual cycle phase was taken into account in the statistical analysis, it was not always possible to separate the main findings of the study considering this variable, especially for the gene analysis. Interestingly, stromal STS secretion was higher during the secretory phase, when compared to glandular STS expression in the nonsecretory phase. Furthermore, DIE lesions expressed more STS protein in the secretory phase than in the nonsecretory phase. These results agree with previously published results (Simpson et al., 1994) and with those from our group (Piccinato et al., 2016b), in which increased STS expression levels were seen in the secretory phase of the cycle. In contrast, other authors have reported that STS expression levels are not altered in the different phases of the menstrual cycle (Šmuc et al., 2007; Colette et al., 2013).

The higher protein expression of ARO was found in stromal EE in relation to DIE, in secretory phase samples, which agrees with the results of ARO tissue protein expression, suggesting that the stromal cells in the endometrium may be the main site of estrogen production. However, some authors have found ARO mRNA and protein expressed preferentially in glandular cells (Aghajanova et al., 2009; Wang et al., 2010). It is known that the endometrial tissue undergoes cyclical changes according to systemic hormonal variation (Foti et al., 2018). However, our findings suggest that the EE may be autonomous with regard to regulation of the ARO pathway, maintaining its expression, and the increased local estrogen production, regardless of systemic hormonal variations. Considering the basic characterization of endometriosis, such as the presence of endometrial tissue in other sites, it is important to understand not only the ectopic behavior of the endometrial-like tissue but also the behavior of that tissue in its place of origin (uterus) during the onset of the disease.

We found high variability among samples in the gene expression of all enzymes in stromal and glandular cell areas. Of note, most samples did not express detectable levels of *CYP19A1* mRNA. The absence of *CYP19A1* in some microdissected biopsies may signify no expression or expression below the detection levels of RT-PCR. In fact, LCM yields small amounts of microdissected material (Matsuzaki et al., 2004) and, consequently, amplification of genes with a low expression such as *CYP19A1* might not reach detectable levels. It is important to consider that the number of microdissected samples for the experiments on gene expression was rather small. Despite having generated interesting primary results, we have to consider them preliminary and subject to confirmation.

## Conclusion

Although the level of the single enzymes in the intracrine machinery varies with apparently no clear association with the localization of endometriotic lesions, a combination of enzymes and site intracrinology may explain site-specific characteristics of the disease. The results of the present study support this idea, as endometriotic sites present, at both gene and protein levels, higher STS and HSD17B1, whereas in EE, there was increased ARO expression. The cellular compartment of *in situ* protein expression seems to emphasize the importance of cell regulation in a paracrine manner. Finally, even though *STS* was the most expressed gene, ARO was the most expressed protein, revealing a complex site-specific system of transcript activation. Although enzyme activity studies are still required to fully understand how estrogen biosynthesis is regulated in the distinct sites where endometriosis occurs, it was possible to substantiate the pronounced gene and protein regulation of *STS* and *HSD17B1*, in endometriotic sites (particularly, in DIE) and of *ARO* in EE. It is apparent that endometriotic lesions at different sites correspond to different facets of the same disease and develop site-dependent intrinsic regulatory mechanisms.

## Data Availability

The original contributions presented in the study are included in the article/Supplementary Material, further inquiries can be directed to the corresponding author.

## Supplementary Material

Supplementary Figure S1 Representative images from STS and HSD17B1 protein expression in both menstrual cycle phases analysed by immunohistochemistry.

Supplementary Figure S2 Relative gene expression in glandular and stromal areas in endometriosis tissue. Gene expression levels of *CYP19A1*, *STS* and *HSD17B1* were analysed by qRT-PCR in eutopic endometrium from endometriosis patients (EE) and lesions. Relative gene expression levels were normalised by *GAPDH*. Bars represent the absolute CT values from each patient individually.
