# Supplementary figures and images for: Site-Specific Regulation of Sulfatase and Aromatase Pathways for Estrogen Production in Endometriosis
Source: Front Mol Biosci. 2022 May 3;9:854991. doi: 10.3389/fmolb.2022.854991 (PMC9110888; doi:10.3389/fmolb.2022.854991)

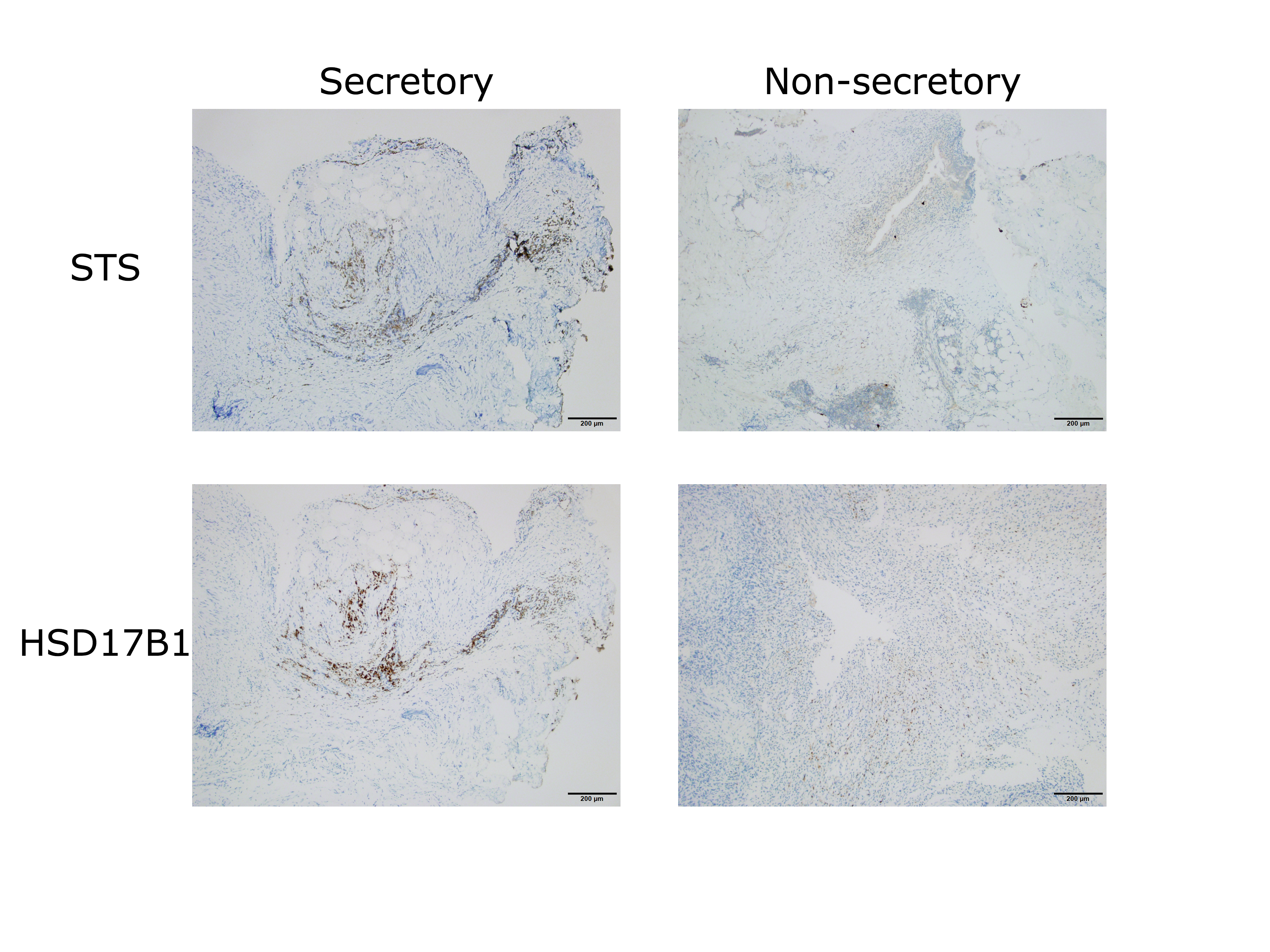

Supplement: Supplementary file 1 [file Image1.JPEG]

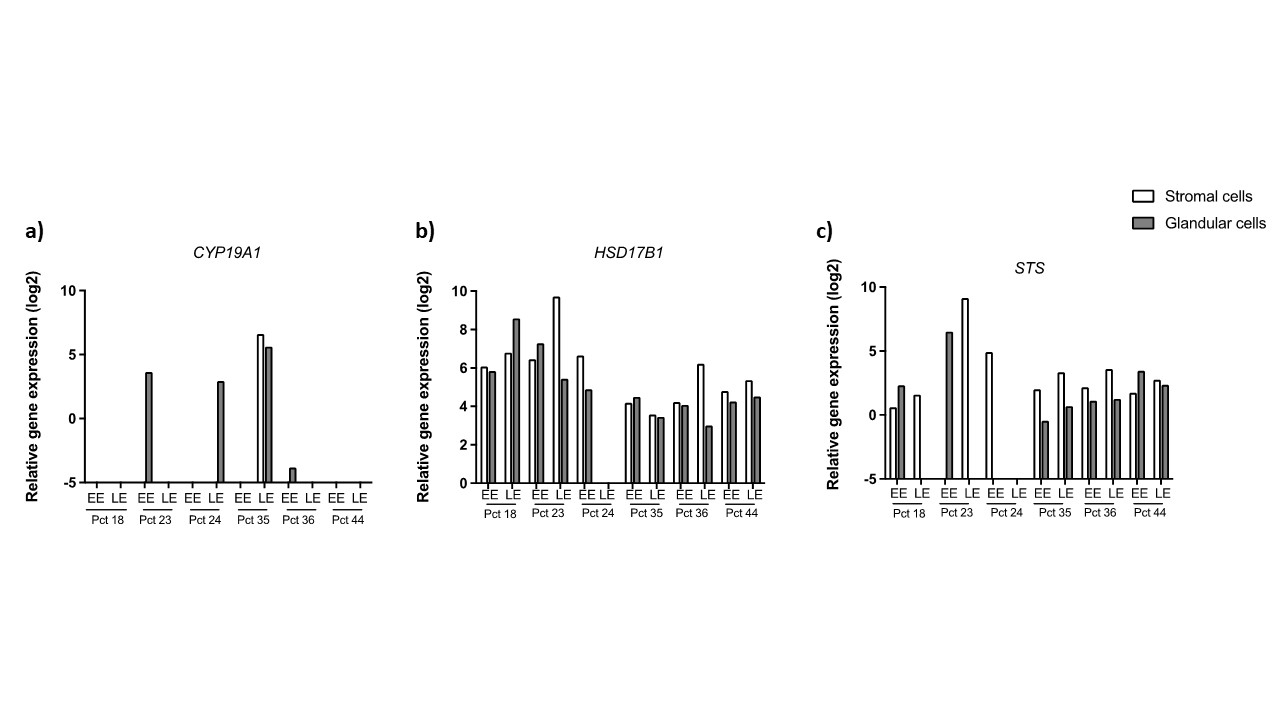

Supplement: Supplementary file 2 [file Image2.JPEG]
